# ^1^H NMR Spectroscopy and MVA to Evaluate the Effects of Caulerpin-Based Diet on *Diplodus sargus* Lipid Profiles

**DOI:** 10.3390/md16100390

**Published:** 2018-10-18

**Authors:** Laura Del Coco, Serena Felline, Chiara Roberta Girelli, Federica Angilè, Laura Magliozzi, Frederico Almada, Biagio D’Aniello, Ernesto Mollo, Antonio Terlizzi, Francesco P. Fanizzi

**Affiliations:** 1 Dipartimento di Scienze e Tecnologie Biologiche ed Ambientali (Di.S.Te.B.A.), Università del Salento, 73100 Lecce, Italy; laura.delcoco@unisalento.it (L.D.C.); chiara.girelli@unisalento.it (C.R.G.); federica.angile@unisalento.it (F.A.); 2 Consorzio Interuniversitario per le Scienze del Mare (CoNISMa), 00196 Roma, Italy; felline@conisma.it (S.F.); antonio.terlizzi@unisalento.it (A.T.); 3 Dipartimento di Biologia, Università degli Studi di Napoli “Federico II”, 80126 Napoli, Italy; laura.magliozzi@unina.it (L.M.); biagio.daniello@unina.it (B.D.); 4 MARE—Marine and Environmental Sciences Centre, ISPA—Instituto Universitário, 1140-041 Lisbon, Portugal; frederico.almada@ispa.pt; 5 Istituto di Chimica Biomolecolare, Consiglio Nazionale delle Ricerche, 80078 Pozzuoli, Italy; ernesto.mollo@icb.cnr.it; 6 Dipartimento di Scienze della Vita, Università degli studi di Trieste, 34127 Trieste, Italy; 7 Department of Biology and Evolution of Marine Organisms, Stazione Zoologica A. Dohrn, 80121 Napoli, Italy

**Keywords:** biological invasion, *Caulerpa cylindracea*, secondary metabolites, *Diplodus sargus*, metabolomics, NMR spectroscopy

## Abstract

The biological invasion of the green algae *Caulerpa cylindracea* represents a serious scientific and public issue in the Mediterranean Sea, essentially due to strong modifications both to habitat structure and native benthic communities. Although alterations in health status and changes in flesh quality of some marine species (dietary exposed to *C. cylindracea*) have been observed, no studies on cause-effect relationships have been carried out. Here, for the first time, through a controlled feeding experiment followed by ^1^H NMR Spectroscopy and multivariate analysis (PCA, OPLS-DA), we showed that caulerpin taken with diet is directly responsible of changes observed in metabolic profile of fish flesh, including alteration of lipid metabolism, in particular with a reduction of ω3 PUFA content. The potential of caulerpin to directly modulate lipid metabolism opens up new questions about causal mechanism triggered by algal metabolite also in view of a possible exploitation in the nutraceutical/medical field.

## 1. Introduction

Biological invasions are, nowadays, a serious environmental issue, representing one of the most important cause of biodiversity loss, with severe ecological, socio-economic and human health repercussions [1,2]. The growing awareness of the problem led to recognize the need of reaching a full understanding of mechanisms by which invasive species (IS) can impact biodiversity and ecosystem functioning. In fact, while their direct effects on biodiversity have been widely investigated, the subtle indirect impacts of molecules (e.g., secondary metabolites) produced by IS on marine ecosystems and communities are almost unexplored [3,4]. Chemical compounds can play a key role in the invasion processes influencing the abundance and distribution of a certain species. For instance, they can act as allelochemicals ensuring the escape from enemy or facilitating the access to resource [3]. Moreover, recent studies provided evidence that molecules from IS enter food chain and have a potential for bioaccumulation phenomena, exerting unexpected and dramatic impacts on native communities [5,6,7]. This is the case of the green alga *Caulerpa cylindracea*, which has heavily affected marine benthic ecosystems [8,9,10] to such an extent that it has been included in the 100 worst invaders for the Mediterranean Sea [11]. Secondary metabolites are suggested to play a crucial role in interspecific interaction processes, thus contributing to determine *C. cylindracea* invasiveness [12,13,14,15]. The most studied secondary metabolites from *C. cylindracea* are the toxic sesquiterpene caulerpenyne, the mixture of ceramides caulerpicin and the alkaloid caulerpin (CAU). All these compounds showed many bioactive properties of potential interest for biotechnological applications [4]. In particular, caulerpin has been showed to accumulate in the lipophilic tissues of the fish *Diplodus sargus* due to their habit to feed on the green algae [16]. Cellular and molecular alterations, metabolic disorders and an impoverishment in nutritional quality of fish flesh were found to correlate with levels of caulerpin in fish tissues [6,7,17,18]. Two potential arguments were put forward for explaining the correlations between the impoverishment of fish nutritional quality and the *C. cylindracea* based-diet. First, a diet exclusively based on *C. cylindracea*, naturally poor in polyunsaturated fatty acids (PUFA), could directly cause a reduction of PUFA in white seabream muscles [17]. Secondly, the observed variations in fatty acids composition could be, at least partially modulated by active metabolites of *C. cylindracea*. The potential role of the algal metabolites as causal factors of altered lipid metabolism in *D. sargus* was suggested by the alteration of the peroxisomal enzymatic activity of acyl-CoA oxidase involved in the β-oxidation of fatty acids and by the activation of gene transcription of peroxisomal proliferator-activated receptor α [6,7]. Moreover, experimental evidence showed dyslipidaemic effects in rats treated with crude *C. cylindracea* extracts [19]. Although the descriptive nature of previous studies and the correlative approach adopted, the definition of causal relationships between a *C. cylindracea* based-diet and the observed effects remained still unclear. Such a gap calls for a complete understanding of the mechanisms triggered by the algal metabolites, even considering their possible impact on the native biota, the public health and the economy. Through a controlled feeding experiment, this study aims at investigating the potential cause-effect relationships between CAU and metabolic alterations in native fish species. In the present work, Proton Nuclear Magnetic Resonance (^1^H NMR) Spectroscopy was applied to analyse the flesh of controlled feeding *D. sargus* specimens and potential variations of their metabolomic profiles were evaluated by multivariate analysis (MVA).

## 2. Results

### NMR Spectroscopy and Multivariate Statistical Analysis (MVA)

**Figure 1:**
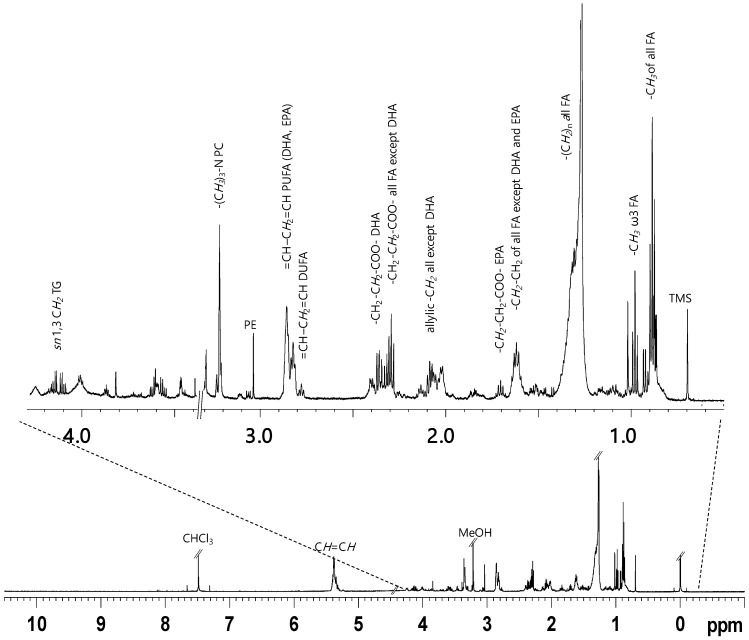
Representative ^1^H NMR spectrum (**down**) and relative expansion (**up**), obtained at 600 MHz of CD_3_OD/CDCl_3_ fish flesh lipid extract.

The lipid extracts of fish muscle were characterized by the presence of lipids (TAGs, PUFA, DUFA, MUFA, SFA) and minor components such as sterols (i.e., cholesterol) and phospholipids (Table 1). Main lipid signals are marked in the ^1^H NMR spectra (Figure 1), corresponding to -C***H***_2_ in alpha and beta-position to the carboxylic acid esters (COOC***H*_2_**C***H*_2_**), unsaturations (CH=CH-C***H*_2_**-CH=CH) of various types of UFA and PUFA (unsaturated and polyunsaturated fatty acids), such as docosahexaenoic (DHA C22:6, ω3), eicosapentaenoic acids (EPA C20:5, ω3) or other PUFA (two and more than two double bonds) of long fatty acids alkyl chain and terminal methyl groups of phospholipids (-CH_3_). The multiplet signals of glycerol moiety of triglycerides (TAGs) appeared at 4.14 and 4.11 ppm (*sn* 1,3), at very low intensities, showing a cross peak correlation with a signal at 5.24 ppm (*sn*2), while the signals in the range of 2.32–2.27 ppm and 1.66–1.57 ppm were assigned to protons of COOC***H*_2_** and COOCH_2_C***H*_2_**, respectively, for all the fatty acids chains, except for DHA (signal at 2.38 ppm, COOC***H*_2_**C***H*_2_**) and EPA (signal at 1.70 ppm COOCH_2_C***H*_2_**). The presence of ω3 PUFA was confirmed by the appearance of triplet at 0.98 ppm due to terminal CH_3_, which is clearly separated from other methyl groups, at 0.88 and 0.87 ppm, due to all other non ω3 fatty acids, such as DUFA (diunsaturated), MUFA (unsaturated) and SFA (saturated fatty acids). The spectra also indicated intense signals in the range 2.88–2.75 ppm due to bisallylic (CH=CH-C***H*_2_**-CH=CH) protons of long alkyl chain fatty acids components. In particular, the multiplet signal at 2.85–2.80 ppm was assigned to bisallylic protons (CH=CH-C***H*_2_**-CH=CH) of DHA and EPA, while bisallylic protons of DUFA appeared at 2.77 ppm, respectively. The presence of partially overlapping singlets at 3.22 and 3.03 ppm are due to the N(CH_3_)_3_ and CH_2_N groups of phospholipids and characteristic of phosphatidylcholine (PC) and phosphatidylethanolamine (PE), respectively. In order to confirm the presence of phospholipids in the extracts, few samples were analysed by ^31^P NMR (spectra not shown). Moreover, signals at 0.68–0.69, 0.92 and 1.01 ppm are the characteristic resonances of cholesterol moieties (CHO).

**Figure 2:**
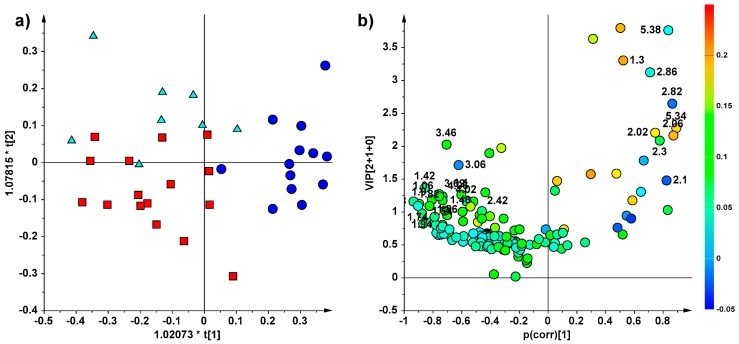
(**a**) OPLS-DA (2 + 1 + 0, R^2^X = 0.696, R^2^Y = 0.563, Q^2^ = 0.362) score plot (triangle, C = control; circle, LD = low dose; box, HD = high dose) obtained from ^1^H NMR lipid extracts for EXP-1 (3 days treatment) and (**b**) relative volcano plot for the model displaying the predictive loadings, using a combination of Variables Influence in Projection (VIP) and the p(corr). Variables are coloured according to the correlation scaled loading (p(corr)). The numbers indicated variables (ppm) in the ^1^H NMR spectra.

**Figure 3:**
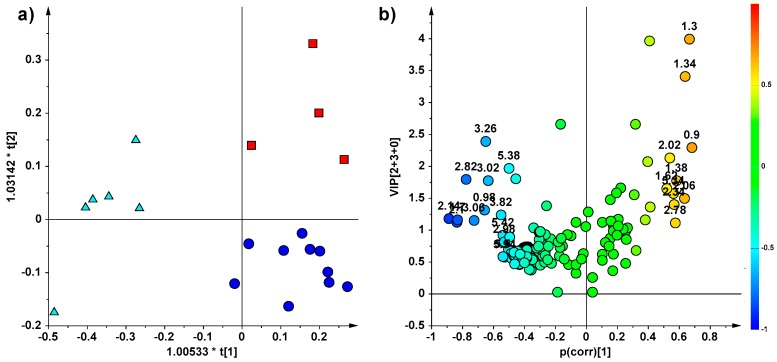
(**a**) OPLS-DA (2 + 3 + 0, R^2^X = 0.912, R^2^Y = 0.798, Q^2^ = 0.462) score plot (triangle, C = control; circle, LD = low dose; box, HD = high dose) obtained from ^1^H NMR lipid extracts for EXP-2 (10 days treatment) and (**b**) relative volcano plot for the model displaying the predictive loadings, using a combination of Variables Influence in Projection (VIP) and the p(corr). Variables are coloured according to the correlation scaled loading (p(corr)). The numbers indicated variables (ppm) in the ^1^H NMR spectra.

Multivariate statistical analyses testing for differences in metabolic profiles among control (C), low dose (LD) and high dose (HD) groups were performed. As a first attempt, in order to identify and display natural groupings of the samples, without imposing any preconception about class membership, a PCA analysis was carried out separately for each experiment (EXP-1 = 37 specimens, EXP-2 = 20 specimens) (Figure S1 in Supplementary Materials). Despite the low number of HD samples in the EXP2, a certain degree of separation was shown in both the two PCA models, especially for LD with respect to C and HD samples. Moreover, a significant CAU effect of fish flesh lipid extracts was observed by applying supervised statistical methods, as OPLS-DA (Figure 2 and Figure 3 for EXP1 and 2, respectively). Specifically, a well descriptive but weakly predictive OPLS-DA model (2 + 1 + 0, R^2^X = 0.696, R^2^Y = 0.563, Q^2^ = 0.362) was obtained for the EXP-1, made of 37 specimens (Figure 2a). LD samples clearly differentiated along the predictive component (the x-axis, t[1]) from the other two groups, (HD and C) samples, whereas the orthogonal component (the y-axis, t[2]) was responsible for C and HD group separation. The study of the variables (NMR signals) responsible for the class separation was observed in the corresponding Volcano Plot (Figure 2b). From this analysis, a high relative content of SFA, MUFA and PUFA (in particular DHA) was observed for C samples, while a high relative content of DUFA were found for LD samples. Finally, HD individuals resulted in a less intense NMR patterns of signals, with respect to other two groups, with no relevant discriminating metabolites.

The OPLS-DA model (2 + 3 + 0, R^2^X = 0.912, R^2^Y = 0.798, Q^2^ = 0.462), obtained from ^1^H NMR lipid extracts of 20 individuals from EXP-2 (Figure 3a), showed a clear-cut separation of C samples, which are clearly distinct along the predictive t[1] axis and clustered on the left-hand side of the graph, from the other two groups of fish fed with the addition of controlled doses of CAU, which, in turn, resulted well separated on the orthogonal component t[2]. Moreover, CAU at low dose seemed to reduce the high natural variability in the metabolic profiles between specimens; a much higher scattering between specimens fed with high or null level of CAU with respect to fish treated with low dose of alkaloid was, indeed, observed (Figure 3a). The molecules (the NMR variables) responsible for the class separation observed in the Volcano Plot (Figure 3b), revealed the presence of a high relative content of PUFA (in particular DHA and EPA) in C samples, whereas a higher relative content of UFA (in particular DUFA) were found in LD and HD samples.

**Figure 4:**
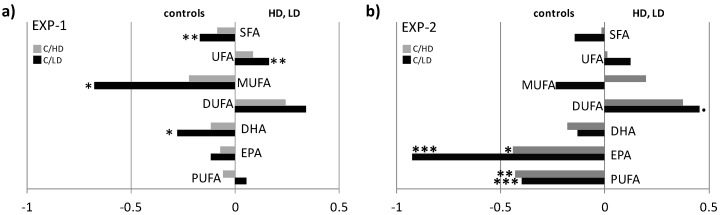
Differences expressed as Fatty Acids percentages for (**a**) EXP-1 (3 days treatment) and (**b**) EXP-2 (10 days treatment), calculated by NMR were represented as Log_2_ fold change (FC) ratio of the corresponding selected signals. Log_2_ (FC) negative values have higher concentration in “controls,” while positive values indicated FA content with higher concentration in LD and HD. Abbreviations: SFA, saturated fatty acids; UFA, unsaturated fatty acids; MUFA, monounsaturated fatty acids; DUFA, diunsaturated fatty acids; DHA, docosahexaenoic acid; EPA, eicosapentaenoic acid, PUFA, polyunsaturated fatty acids; Statistical significance (One way-ANOVA test with Multiple Comparisons of Means, Tukey′s honestly significant difference, HSD post hoc test), Signif. codes: 0 ‘***’ 0.001 ‘**’ 0.01 ‘*’ 0.05 ‘**·**’ 0.1 ‘ ‘ 1. (Tables S7 and S8).

Fatty acids (FA) percentages were calculated by the integration of the corresponding selected NMR signals and differences were represented as Log_2_ fold change (FC) ratio [20,21]. Signals at 0.87–0.88 ppm (-C***H*_3_** of all except ω3 FA), 1.67–1.74 ppm (COOCH_2_C***H*_2_** of EPA), 1.99–2.17 ppm (-CH=CH-C***H*_2_** of all FA except DHA), 2.27–2.35 ppm (COOC*H_2_* of all FA except DHA), 2.38 ppm (COOC***H*_2_**C***H*_2_** of DHA) and 2.77 ppm (CH=CH-C***H*_2_**-CH=CH of DUFA), were selected and integrated for quantification of FA percentage [22,23,24,25]. Obtained values were calculated as Log_2_ fold change (FC) ratio of the corresponding selected signals and One way-ANOVA (with Multiple Comparisons of Means, Tukey’s honestly significant difference, HSD post hoc) test was performed on mean differences (Tables S1–S6 in Supplementary Materials). Looking at the EXP-1 fish flesh lipid extracts, statistically significant high levels of SFA, MUFA and DHA were found in C with respect to LD samples, whereas these last showed a significant increase of UFA and DUFA. On the other hand, no significant differences in FA composition were observed between C and HD groups (Figure 4a). Differently from EXP-1, samples from EXP-2 had statistically significant high levels of PUFA (in particular EPA) in C with respect to both LD and HD samples, with an increase of DUFA content in LD class (Figure 4b).

## 3. Discussion

The ^1^H NMR-based controlled feeding experiments revealed that CAU causes some modifications of metabolomic lipid profiles of juvenile *D. sargus*, with more pronounced differences observed after the longest period of exposure (10 days, EXP-2). A general decrease of PUFA (especially of EPA and DHA) and an increase of UFA, were found in fish fed with food enriched with CAU, supporting our previous results, reported in Felline et al. [17]. On the other hand, a decrease of the percentage of eicosapentaenoic, docosahexaenoic and arachidonic acids was found in wild fish naturally feeding on *C. cylindracea* [17]. Differences in lipid composition of muscle and liver were attributed, at the time, to the change from an omnivorous feeding habit to a diet almost exclusively based on *C. cylindracea*, depriving fish from those essential fatty acids which they cannot biosynthesize [17,26]. For the first time, the present study showed the causal relationship between algal metabolite and the alteration of lipid profile in fish. Interestingly, differences were clearly observed between C and LD with respect to C and HD. For both the statistical models studied, indeed, no evidence of significant differences was found between C and HD groups, even if the patterns of metabolic profile remained the same, with a general reduction of PUFA and an increase in UFA (and DUFA) in treated fish with respect to the controls. These patterns could be explained with the gradually loss of appetite and voracity, due to an inadequate food intake, especially for the individuals fed with the highest dosage levels [27]. CAU shows structural similarity to endogenous indolamines that modulate animal behaviour and a reduction of aggressive behaviour was described in fish exposed to CAU high dose [28]. Moreover, as already reported in literature, this metabolite exerts a sedative effect via pathways involving serotonin 5-HT3 receptors [29]. Therefore, the lower difference in metabolic profiles observed for HD and C individuals may be also explained by the onset of anorexigenic effect with the consequent reduction of the amount of CAU ingested. Although direct effect of CAU on lipid composition of fish fillet has been demonstrated, it remains still unclear which are the cellular or molecular processes triggered by CAU and involved in the observed alterations. Our previous results indicated that a CAU-enriched diet affect the activity of acyl-CoA oxidase, a peroxisomal enzyme involved in the β-oxidation of fatty acids and the gene expression of peroxisomal proliferator-activated receptor α [6,7], suggesting the potential involvement of algal metabolites in the peroxisomal proliferator-activated receptors (PPARs). In particular, CAU could act as PPAR-α agonist, a member of the nuclear hormone receptor superfamily mediating peroxisome proliferation and the increase in fatty acyl-CoA oxidase activity, that it is known to bind with several high affinity xenobiotic ligands, such as hypolipidaemic agents, plasticizers, solvents and herbicides [30]. Moreover, it is known that treatments with PPAR-α agonists tend to increase expression of peroxisomal ROS-generating enzymes without increasing catalase, resulting in an imbalance between ROS production and elimination [31]. Similar pattern was found in Gorbi et al. [7], where fish with high level of CAU were characterized by both a significant increase in AOX activity and in gene expression of peroxisomal proliferator-activated receptor α but not significant increase in catalase activity was observed. Since lipid metabolism plays a multifunctional role in various mechanisms such as long-term energy storage, intercellular and intracellular signalling and membrane homeostasis, this work sheds light on a critical aspect of biological invasions with implication on health of local fish populations and related economies. Abnormalities in lipid metabolism could reduce growth and development, impair fertility and cause several pathologies or even death [32,33]; therefore, preserving lipid homeostasis is crucial for an organism’s survival, health and reproduction [34]. In addition to detrimental effects on the fish health, the reduction in the levels and quality of FA could seriously damage fishery economy by lowering fillet nutritional quality of Mediterranean fish species, considering also that effects observed in *D. sargus* could manifest in other commercial fish species eating this invasive algal species [16].

## 4. Materials and Methods

### 4.1. Sample Collection and Experiment Design

Fish specimens analysed are the same collected and studied in the parallel work focusing on behavioural changes modulated by dietary caulerpin (CAU). The study was specifically approved by the Animal Care and Use Committee of ISPA-Instituto Universtário (ORBEA-ISPA; Permit Number: 01-2017). It did not involve endangered or protected species and was conducted under the supervision of an accredited expert in laboratory animal science (following FELASA category C recommendations). Permission for capturing fish at the field site was granted by the Portuguese Environmental Agency (APA) and by local authorities (Cascais Environmental Agency—Cascais Ambiente—and Coast Guard—Capitania de Cascais) [28]. Juvenile fish were captured with hand nets in 2015 near Cascais, in the central Portugal region and transferred in constantly aerated tanks at ISPA-IU. Fish were initially monitored, measured and weighed, then stored in containers and randomly housed in sea water aquaria. All individuals were juveniles and belonged to the same size class, with a mean body weight of 1.82 ± 0.1 g and a mean standard length of 3.99 ± 0.1 cm. Physicochemical variables of sea water were maintained constant throughout the experiment at following settings: temperature, 20–22 °C; dissolved oxygen, 7 mg L^−1^; pH 7–8; salinity 33–35 g L^−1^; NH_4_ and NO_2_ never exceeding 0.5 mg L^−1^. In order to investigate potential effects of algal metabolite on fish lipid metabolism, *D. sargus* individuals were fed with 0.25 g of commercial pellet enriched with CAU at three different concentrations: Control groups (C), with fish fed with food without CAU, Low Dose groups (LD), in which food was implemented with CAU at natural estimated levels in *C. cylindracea* (0.1 mg g^−1^) and High Dose groups (HD), with a dose of CAU ten-fold higher (1.0 mg g^−1^). Moreover, two different group experiments were performed, with 37 and 20 individuals respectively, randomly assigned to tanks of Control (C) and Low Dose (LD) and High Dose (HD) of CAU. In the first group experiment (EXP-1; 37 individuals, C = 8, LD = 13, HD = 16 specimens) the acclimation, treatment and post-treatment phases were set at 3 days each, whereas for the second group experiment (EXP-2; 20 individuals, C = 6, LD = 10, HD = 4 specimens) the acclimation and treatment period was set at 10 days and the post-treatment phases lasted only 3 days. Prior to proceeding with the dissection of muscle, fish were euthanized with an overdose of MS-222 (Pharmaq, Oslo, Norway). Muscle tissues were frozen in liquid nitrogen and maintained at −80 °C till processed for chemical analyses.

### 4.2. Sample Preparation for NMR Analysis

Muscle samples were prepared according to a modified Bligh and Dyer two-step method [35,36]. Fish tissue (~150 mg) was added of 400 μL methanol and 200 μL deionized filtered water; then, the sample was homogenized with a stainless-steel bead in the TissueLyser for 2–3 min at 25 Hz. Chloroform (400 μL) and deionized filtered water (200 μL) were added to homogenate. The solution was mixed and placed on ice for 10 min before centrifugation at 10,000 rpm for 20 min at 4 °C. The hydrophilic and lipophilic phases were separated and dried by SpeedVac concentrator. Muscle lipid fractions were dissolved in 700 μL CD_3_OD/CDCl_3_ (1:2 mix) and transferred to a 5-mm NMR tube. Hydrophilic extracts were stored at −20 °C for further NMR analysis.

### 4.3. NMR Measurements

All measurements were performed on a Bruker Avance III 600 Ascend NMR spectrometer (Bruker, Hamburg, Germany) operating at 600.13 MHz for ^1^H observation, equipped with a z axis gradient coil and automatic tuning-matching (ATM). Experiments were acquired at 300 K in automation mode after loading individual samples on a Bruker Automatic Sample Changer, interfaced with the software IconNMR (Bruker). For each lipid extract a one-dimensional experiment (zg Bruker pulse program) was run with 64 scans, 64 K time domain, spectral width 20.0276 ppm (12,019.230 Hz), 3 s delay, p1 10 µs and 2.73 s acquisition time. All spectra were referenced to the tetramethylsilane (TMS) signal (δ = 0.00 ppm). ^31^P NMR spectra (zg0pg Bruker pulse program) were acquired with a spectral width of 50.1172 ppm (12,175.324 Hz), p1 11 µs, 3 s delay and 1.34 s acquisition time and referenced to H_3_PO_4_ as external standard. The metabolites were assigned on the basis of 2D NMR spectra analysis (2D ^1^H JRES, ^1^H COSY, ^1^H-^13^C HSQC and HMBC) and comparison with published data [18,23,37,38].

### 4.4. Data Analysis

NMR spectra were processed using Topspin 2.1 and using Amix 3.9.13 (Bruker, Biospin, Italy) for simultaneous visual inspection and the successive bucketing process: the ^1^H NMR spectra of lipid extract were segmented in rectangular buckets of fixed 0.04 ppm width and integrated. The spectral regions between 7.8–7.2, 4.6–4.2 and 3.4–3.3 ppm were discarded because of the residual peak of solvents (chloroform and methanol signals). The resulting data sets resulted in a matrix, made of the bucketed ^1^H NMR spectra values (columns) measured each sample, that is, the lipid fish extract (rows). The Pareto scaling procedure was applied to the data, which was performed by dividing the mean-centred data by the square root of the standard deviation [39]. The data table generated with all the spectra were submitted to multivariate data analysis (MVA). Both unsupervised (principal component analysis, PCA) and supervised (orthogonal partial least squares discriminant analysis, OPLS-DA) pattern recognition methods were performed to examine the intrinsic variation in the data [40,41,42]. The robustness and predictive ability of the statistical models for discrimination purposes were tested by cross-validation default method (7-fold) and further evaluated with permutation test (400 permutations) of SIMCA 14 software, (Sartorius Stedim Biotech, Umeå, Sweden) [42]. The R^2^ and Q^2^ are the two parameters that describe the goodness of the models. The former (R^2^) explains the total variations in the data, whereas the latter (Q^2^) is an internal cross validation parameter, which indicates the predictability of the model [43]. By ^1^H NMR Spectroscopy, fatty acids composition was quantified by analysing the integrals of selected distinctive unbiased NMR signals [23,24,25]. Differences were represented as Log_2_ fold change (FC) ratio of the calculated average intensities of the corresponding selected signals [20,21]. Results were validated by the analysis of variance (One Way-ANOVA) with Tukey’s honestly significant difference (HSD) post hoc test, using the R statistical environment, Version 3.4.1, on a 64bit Windows machine [44]. The levels of statistical significance were at least at *p*-values < 0.05 with 95% confidence level.

### 4.5. Chemicals

All chemical reagents for analysis were of analytical grade. CDCl_3_, CD_3_OD (99.8 atom%D), TMS (0.03 *v*/*v*%) were purchased from Armar Chemicals (Döttingen, Switzerland).

## 5. Conclusions

In conclusion, the present study provided the evidence of a direct effect of CAU on fish flesh lipid metabolic profile, in particular with a significant loss of PUFA in fish fed for a long period of exposure (10 days) with a CAU-enriched diet. Nevertheless, molecular mechanisms responsible for the fatty acids changes still remained to be completely elucidated. Actually, longer period experiments are in progress to further deepen impacts of algal metabolite on fish. In particular, laboratory in vitro, ex vivo, in vivo assays and in silico models’ validation studies are ongoing to fully assess the mechanisms of action of CAU, in order to clarify the complex indirect effects of pest metabolites on marine biodiversity at the species and ecosystem level.

## Figures and Tables

**Table 1:** Chemical shifts ^1^H (ppm) and assignments of metabolite resonances in the ^1^H NMR spectrum of muscle lipid extract (CHO, Cholesterol, DHA, Docosahexaenoic acid, EPA, Eicosapentaenoic acid, SFA, Saturated Fatty Acids, MUFA, Monounsaturated Fatty Acids, DUFA, Diunsaturated Fatty Acids, PUFA, Polyunsaturated Fatty Acids, TAGs, Triacilglycerols, PC, phosphatidylcholine, PE, phosphatidylethanolamine).

Compound	Assignment	^1^H (ppm, multiplicity)
CHO	-C***H*_3_**-18 -C***H*_3_**-21 -C***H*_3_**-19	0.68–0.69 (s) 0.92 (d) 1.01 (s)
All **FA** (SFA, MUFA, DUFA) except ω3 FA	-C***H*_3_**	0.87–0.88 (t) *
ω3 PUFA	-C***H*_3_**	0.98 (t) *
All fatty acids	-(C***H*_2_**)_n_	1.22–1.34 (m)
All fatty chains except DHA and EPA	COOCH_2_C***H*_2_**	1.57–1.66 (m)
EPA	COOCH_2_C***H*_2_**	1.67–1.74 (m) *
All fatty acids except DHA	-CH=CH-C***H*_2_**	1.99–2.17 (m) *
All fatty acids except DHA	COOC***H*_2_**	2.27–2.35 (t) *
DHA	COOC***H*_2_**C***H*_2_**	2.38 (dd) *
DUFA	CH=CH-C***H*_2_**-CH=CH	2.77 (t) *
PUFA (DHA, EPA)	CH=CH-C***H*_2_**-CH=CH	2.80–2.85 (t)
PE	-C***H*_2_**-N	3.03 (s)
PC	-(C***H*_3_**)_3_-N	3.22 (s)
TAGs	C***H*_2_** (*sn*1,3) C***H*** (*sn*2)	4.11–4.14 (dd) 5.24 (m)
All FA	C***H***=C***H***	5.28–5.43 (m)

* Signals selected for quantification of FA percentage.

## Supplementary Materials

The following are available online at http://www.mdpi.com/1660-3397/16/10/390/s1, Figure S1. (a) PCA (4 components, R^2^X = 0.80, Q^2^ = 0.70) score plot obtained from ^1^H NMR lipid extracts for experiment 1 (3 days treatment); (b) PCA (4 components, R^2^X = 0.89, Q^2^ = 0.74) score plot for experiment 2 (10 days treatment); Table S1. Fatty acid percentage calculated by integration of unbiased signals in the ^1^H NMR spectra of lipid extracts for EXP-1 samples; Table S2. Ratio calculated from percentage of FA values; Table S3. Fold change (FC) values; Table S4. Fatty acid percentage calculated by integration of unbiased signals in the ^1^H NMR spectra of lipid extracts for EXP-2 samples; Table S5. Ratio calculated from percentage of FA values; Table S6. Fold change (FC) values; Table S7: One-way Anova results for EXP-1 samples; Table S8: One-way Anova results for EXP-2 samples.

## Abbreviations

IS	Invasive Species
C	Control
CAU	Caulerpin
CHO	Cholesterol
DHA	Docosahexaenoic Acid
DUFA	Diunsaturated Fatty Acids
EPA	Eicosapentaenoic Acid
HD	High Dose
LD	Low Dose
MVA	Multivariate Analysis
MUFA	Monounsaturated Fatty Acids
OPLS-DA	Orthogonal Partial Least Squares Discriminant Analysis
PCA	Principal Component Analysis
PC	Phosphatidylcholine
PE	Phosphatidylethanolamine
PLS-DA	Partial Least Squares Discriminant Analysis
PUFA	Polyunsaturated Fatty Acids
SFA	Saturated Fatty Acids
TAGs	Triacilglycerols
UFA	Unsaturated Fatty Acids
