# Supplementary material for: 1H NMR Spectroscopy and MVA to Evaluate the Effects of Caulerpin-Based Diet on Diplodus sargus Lipid Profiles
Source: Mar Drugs. 2018 Oct 18;16(10):390. doi: 10.3390/md16100390 (PMC6213232; doi:10.3390/md16100390)
Supplement: Supplementary file 1 [file marinedrugs-16-00390-s001.pdf]

# Supplementary Material

# $^{1}$H NMR Spectroscopy and MVA to Evaluate the Effects of Caulerpin-Based Diet on *Diplodus Sargus* Lipid Profiles

**Laura Del Coco [1,†], Serena Felline [2,†], Chiara Roberta Girelli [1], Federica Angilè [1], Laura Magliozzi [3], Frederico Almada [4], Biagio D'Aniello [3], Ernesto Mollo [5], Antonio Terlizzi [2,6,7] and Francesco P. Fanizzi [1,*]**

1 Dipartimento di Scienze e Tecnologie Biologiche ed Ambientali (Di.S.Te.B.A.), Università del Salento, 73100 Lecce, Italy; laura.delcoco@unisalento.it (L.D.C.); chiara.girelli@unisalento.it (C.R.G.); federica.angile@unisalento.it (F.A.)
2 Consorzio Interuniversitario per le Scienze del Mare (CoNISMa), 00196 Roma, Italy; felline@conisma.it (S.F.); antonio.terlizzi@unisalento.it (A.T.)
3 Dipartimento di Biologia, Università degli Studi di Napoli "Federico II" 80126 Napoli, Italy; laura.magliozzi@unina.it (L.M.); biagio.daniello@unina.it (B.D.A.)
4 MARE—Marine and Environmental Sciences Centre, ISPA—Instituto Universitário, 1140-041 Lisbon, Portugal; frederico.almada@ispa.pt
5 Istituto di Chimica Biomolecolare, Consiglio Nazionale delle Ricerche, 80078 Pozzuoli, Napoli, Italy; ernesto.mollo@icb.cnr.it
6 Dipartimento di Scienze della Vita, Università degli studi di Trieste, 34127 Trieste, Italy;
7 Department of Biology and Evolution of Marine Organisms, Stazione Zoologica A. Dohrn, 80121 Napoli, Italy
* Correspondence: fp.fanizzi@unisalento.it; Tel.: +39-0832-29265; Fax: +39-0832-298626
† These authors contributed equally to this work.

---

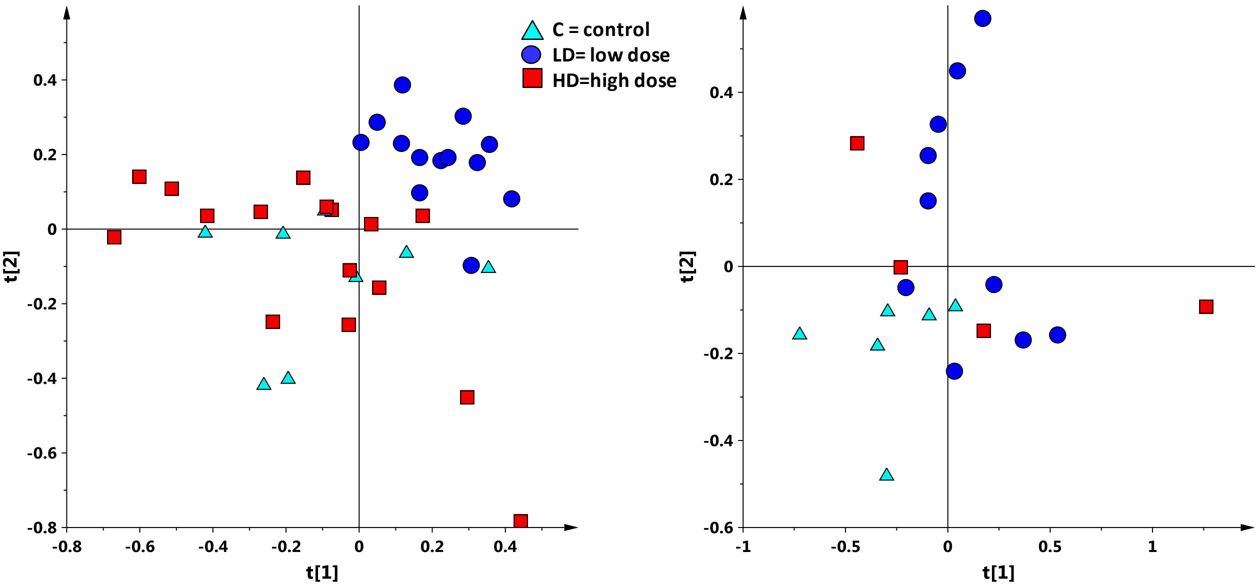


(a) (b)

**Figure S1.** (**a**) PCA (4 components, $R^2X$=0.80, $Q^2$=0.70) score plot obtained from $^{1}$H NMR lipid extracts for experiment 1 (3 days treatment); (**b**) PCA (4 components, $R^2X$=0.89, $Q^2$=0.74) score plot for experiment 2 (10 days treatment).

**FOLD CHANGE EXP-1 (37 samples)**

**Table S1.** Fatty acid percentage calculated by integration of unbiased signals in the $^{1}$H NMR spectra of lipid extracts for EXP-1 samples.

| | %PUFA | %EPA | %DHA | %DUFA | %MUFA | %UFA | %SFA |
|---|---|---|---|---|---|---|---|
| C | 23.23 | 3.75 | 12.25 | 6.32 | 22.37 | 48.08 | 51.92 |
| HD | 22.31 | 3.57 | 11.29 | 7.47 | 19.20 | 51.02 | 48.98 |
| LD | 24.13 | 3.46 | 10.11 | 8.01 | 14.00 | 53.86 | 46.14 |

**Table S2.** Ratio calculated from percentage of FA values.

| | %PUFA | %EPA | %DHA | %DUFA | %MUFA | %UFA | %SFA |
|---|---|---|---|---|---|---|---|
| C/LD | 0.963 | 1.083 | 1.212 | 0.789 | 1.598 | 0.893 | 1.125 |
| C/HD | 1.041 | 1.050 | 1.085 | 0.845 | 1.165 | 0.942 | 1.060 |

**Table S3.** Fold change (FC) values.

| FC | %PUFA | %EPA | %DHA | %DUFA | %MUFA | %UFA | %SFA |
|---|---|---|---|---|---|---|---|
| C/LD | 0.054 | -0.115 | -0.277 | 0.342 | -0.676 | 0.164 | -0.170 |
| C/HD | -0.058 | -0.070 | -0.117 | 0.243 | -0.221 | 0.085 | -0.084 |

**FOLD CHANGE EXP-2 (20 samples)**

**Table S4.** Fatty acid percentage calculated by integration of unbiased signals in the $^{1}$H NMR spectra of lipid extracts for EXP-2 samples.

| | %PUFA | %EPA | %DHA | %DUFA | %MUFA | %UFA | %SFA |
|---|---|---|---|---|---|---|---|
| C | 16.88 | 1.77 | 9.22 | 12.44 | 14.70 | 55.99 | 44.01 |
| HD | 16.52 | 2.48 | 8.89 | 11.78 | 19.88 | 51.82 | 48.18 |
| LD | 22.28 | 3.37 | 10.08 | 9.07 | 17.31 | 51.33 | 48.67 |

**Table S5.** Ratio calculated from percentage of FA values.

| | %PUFA | %EPA | %DHA | %DUFA | %MUFA | %UFA | %SFA |
|---|---|---|---|---|---|---|---|
| C/LD | 1.320 | 1.902 | 1.094 | 0.729 | 1.178 | 0.917 | 1.106 |
| C/HD | 1.349 | 1.359 | 1.134 | 0.770 | 0.871 | 0.991 | 1.010 |

**Table S6.** Fold change (FC) values.

| FC | %PUFA | %EPA | %DHA | %DUFA | %MUFA | %UFA | %SFA |
|---|---|---|---|---|---|---|---|
| C/LD | -0.401 | -0.9278 | -0.130 | 0.455 | -0.236 | 0.125 | -0.145 |
| C/HD | -0.432 | -0.442 | -0.181 | 0.377 | 0.199 | 0.014 | -0.014 |

**Table S7. One-way Anova results for EXP-1 samples.**

```
> AnovaModel.2 <- aov(X.PUFA ~ trattamento, data=dsE7_TOT)

> summary(AnovaModel.2)
            Df Sum Sq Mean Sq F value Pr(>F)
trattamento  2  23.69  11.847   2.127  0.135
Residuals   34 189.33   5.568
```

```
> with(dsE7_TOT, numSummary(X.PUFA, groups=trattamento, statistics=c("mean",
+    "sd")))
       mean       sd data:n
C  23.23309 2.519209      8
HD 22.31423 2.485671     16
LD 24.12908 2.086125     13

Fit: aov(formula = X.PUFA ~ trattamento, data = dsE7_TOT)

Linear Hypotheses:
            Estimate Std. Error t value Pr(>|t|)
HD - C == 0   -0.9189     1.0218  -0.899    0.643
LD - C == 0    0.8960     1.0604   0.845    0.677
LD - HD == 0   1.8148     0.8811   2.060    0.113
(Adjusted p values reported -- single-step method)

> AnovaModel.5 <- aov(X.EPA ~ trattamento, data=dsE7_TOT)

> summary(AnovaModel.5)
            Df Sum Sq Mean Sq F value Pr(>F)
trattamento  2  0.410  0.2051   0.967   0.39
Residuals   34  7.211  0.2121

> with(dsE7_TOT, numSummary(X.EPA, groups=trattamento, statistics=c("mean", "sd")))
       mean        sd data:n
C  3.751333 0.3737482      8
HD 3.572989 0.4962436     16
LD 3.463528 0.4600527     13

Fit: aov(formula = X.EPA ~ trattamento, data = dsE7_TOT)

Linear Hypotheses:
            Estimate Std. Error t value Pr(>|t|)
HD - C == 0   -0.1783     0.1994  -0.894    0.646
LD - C == 0   -0.2878     0.2069  -1.391    0.355
LD - HD == 0  -0.1095     0.1720  -0.637    0.800
(Adjusted p values reported -- single-step method)



> AnovaModel.6 <- aov(X.DHA ~ trattamento, data=dsE7_TOT)

> summary(AnovaModel.6)
            Df Sum Sq Mean Sq F value Pr(>F)
trattamento  2  23.92  11.961   5.034 0.0122 *
Residuals   34  80.78   2.376
---
Signif. codes:  0 '***' 0.001 '**' 0.01 '*' 0.05 '.' 0.1 ' ' 1
```

```
> with(dsE7_TOT, numSummary(X.DHA, groups=trattamento, statistics=c("mean", "sd")))
       mean       sd data:n
C  12.24687 1.093859      8
HD 11.28827 1.842136     16
LD 10.10589 1.338741     13


Multiple Comparisons of Means: Tukey Contrasts


Fit: aov(formula = X.DHA ~ trattamento, data = dsE7_TOT)

Linear Hypotheses:
             Estimate Std. Error t value Pr(>|t|)
HD - C == 0   -0.9586     0.6675  -1.436   0.3326
LD - C == 0   -2.1410     0.6927  -3.091   0.0107 *
LD - HD == 0  -1.1824     0.5756  -2.054   0.1141
---
Signif. codes:  0 '***' 0.001 '**' 0.01 '*' 0.05 '.' 0.1 ' ' 1
(Adjusted p values reported -- single-step method)


> with(dsE7_TOT, numSummary(X.DUFA, groups=trattamento, statistics=c("mean",
+   "sd")))
       mean       sd data:n
C  6.315064 1.908038      8
HD 7.473351 2.257739     16
LD 8.005193 1.440296     13


         Simultaneous Tests for General Linear Hypotheses

Multiple Comparisons of Means: Tukey Contrasts


Fit: aov(formula = X.DUFA ~ trattamento, data = dsE7_TOT)

Linear Hypotheses:
             Estimate Std. Error t value Pr(>|t|)
HD - C == 0    1.1583     0.8363   1.385    0.358
LD - C == 0    1.6901     0.8679   1.947    0.140
LD - HD == 0   0.5318     0.7212   0.737    0.742


> AnovaModel.8 <- aov(X.MUFA ~ trattamento, data=dsE7_TOT)

> summary(AnovaModel.8)
            Df Sum Sq Mean Sq F value Pr(>F)
trattamento  2  383.5  191.75   3.892 0.0301 *
```

```
Residuals   34 1675.2   49.27
---
Signif. codes:  0 '***' 0.001 '**' 0.01 '*' 0.05 '.' 0.1 ' ' 1

> with(dsE7_TOT, numSummary(X.MUFA, groups=trattamento, statistics=c("mean",
+   "sd")))
       mean       sd data:n
C  22.37170 8.244414      8
HD 19.19655 8.009060     16
LD 14.00173 4.446400     13
```

Multiple Comparisons of Means: Tukey Contrasts

```
Fit: aov(formula = X.MUFA ~ trattamento, data = dsE7_TOT)

Linear Hypotheses:
             Estimate Std. Error t value Pr(>|t|)
HD - C == 0    -3.175      3.039  -1.045   0.5529
LD - C == 0    -8.370      3.154  -2.654   0.0312 *
LD - HD == 0   -5.195      2.621  -1.982   0.1312
---
Signif. codes:  0 '***' 0.001 '**' 0.01 '*' 0.05 '.' 0.1 ' ' 1
(Adjusted p values reported -- single-step method)



> AnovaModel.9 <- aov(X.UFA ~ trattamento, data=dsE7_TOT)

> summary(AnovaModel.9)
            Df Sum Sq Mean Sq F value  Pr(>F)
trattamento  2  169.4   84.72   5.318 0.00978 **
Residuals   34  541.6   15.93
---
Signif. codes:  0 '***' 0.001 '**' 0.01 '*' 0.05 '.' 0.1 ' ' 1

> with(dsE7_TOT, numSummary(X.UFA, groups=trattamento, statistics=c("mean", "sd")))
       mean       sd data:n
C  48.08015 5.023999      8
HD 51.01588 4.292175     16
LD 53.86400 2.717480     13
```

Multiple Comparisons of Means: Tukey Contrasts

```
Fit: aov(formula = X.UFA ~ trattamento, data = dsE7_TOT)

Linear Hypotheses:
```

```
            Estimate Std. Error t value Pr(>|t|)
HD - C == 0     2.936      1.728   1.699  0.21910
LD - C == 0     5.784      1.794   3.225  0.00756 **
LD - HD == 0    2.848      1.490   1.911  0.14999
---
Signif. codes:  0 '***' 0.001 '**' 0.01 '*' 0.05 '.' 0.1 ' ' 1
(Adjusted p values reported -- single-step method)


> AnovaModel.10 <- aov(X.SFA ~ trattamento, data=dsE7_TOT)

> summary(AnovaModel.10)
            Df Sum Sq Mean Sq F value  Pr(>F)
trattamento  2  169.4   84.72   5.318 0.00978 **
Residuals   34  541.6   15.93
---
Signif. codes:  0 '***' 0.001 '**' 0.01 '*' 0.05 '.' 0.1 ' ' 1


> with(dsE7_TOT, numSummary(X.SFA, groups=trattamento, statistics=c("mean", "sd")))
       mean       sd data:n
C  51.91985 5.023999      8
HD 48.98412 4.292175     16
LD 46.13600 2.717480     13

         Simultaneous Tests for General Linear Hypotheses

Multiple Comparisons of Means: Tukey Contrasts


Fit: aov(formula = X.SFA ~ trattamento, data = dsE7_TOT)

Linear Hypotheses:
            Estimate Std. Error t value Pr(>|t|)
HD - C == 0    -2.936      1.728  -1.699  0.21908
LD - C == 0    -5.784      1.794  -3.225  0.00761 **
LD - HD == 0   -2.848      1.490  -1.911  0.15007
---
Signif. codes:  0 '***' 0.001 '**' 0.01 '*' 0.05 '.' 0.1 ' ' 1
(Adjusted p values reported -- single-step method)
```

**Table S8. One-way Anova results for EXP-2 samples.**

```
         Simultaneous Tests for General Linear Hypotheses

Multiple Comparisons of Means: Tukey Contrasts

> with(DSL_M25_simca, numSummary(PUFA, groups=trattamento, statistics=c("mean", "sd")))
```

```
     mean       sd data:n
C 22.28495 1.526364      6
HD 16.51727 3.000546      4
LD 16.87872 2.283117     10

Fit: aov(formula = X.PUFA ~ trattamento, data = DSL_M25_simca)

Linear Hypotheses:
          Estimate Std. Error t value Pr(>|t|)
HD - C == 0  -5.7677     1.4482  -3.983  0.00265 **
LD - C == 0  -5.4062     1.1586  -4.666  < 0.001 ***
LD - HD == 0   0.3615     1.3273   0.272  0.95973
---

> with(DSL_M25_simca, numSummary(EPA, groups=trattamento, statistics=c("mean", "sd")))
     mean        sd data:n
C 3.366663 0.5306937      6
HD 2.478220 0.5833759      4
LD 1.769966 0.2715293     10

Fit: aov(formula = EPA ~ trattamento, data = DSL_M25_simca)

Linear Hypotheses:
          Estimate Std. Error t value Pr(>|t|)
HD - C == 0  -0.8884     0.2753  -3.227   0.0128 *
LD - C == 0  -1.5967     0.2203  -7.249   <0.001 ***
LD - HD == 0  -0.7083     0.2523  -2.807   0.0307 *


> AnovaModel.4 <- aov(DHA ~ trattamento, data=DSL_M25_simca)
           Df Sum Sq Mean Sq F value Pr(>F)
trattamento  2   4.17   2.083   0.449  0.646
Residuals   17  78.87   4.639

> with(DSL_M25_simca, numSummary(DHA, groups=trattamento,
+   statistics=c("mean", "sd")))
      mean       sd data:n
C 10.081935 1.886349      6
HD  8.891368 1.876738      4
LD  9.215016 2.369036     10

Multiple Comparisons of Means: Tukey Contrasts


Fit: aov(formula = X.DHA ~ trattamento, data = DSL_M25_simca)

Linear Hypotheses:
          Estimate Std. Error t value Pr(>|t|)
HD - C == 0  -1.1906     1.3903  -0.856    0.672
```

```
LD - C == 0  -0.8669     1.1123  -0.779    0.719
LD - HD == 0   0.3236     1.2743   0.254    0.965
(Adjusted p values reported -- single-step method)
```

```
> AnovaModel.5 <- aov(DUFA ~ trattamento, data=DSL_M25_simca)

> summary(AnovaModel.5)
            Df Sum Sq Mean Sq F value Pr(>F)
trattamento  2  43.69  21.844   2.827 0.0871 .
Residuals   17 131.34   7.726
---

> with(DSL_M25_simca, numSummary(DUFA, groups=trattamento,
+   statistics=c("mean", "sd")))
       mean       sd data:n
C  9.069537 1.927444      6
HD 11.781788 5.177614      4
LD 12.436267 1.895603     10

Fit: aov(formula = DUFA ~ trattamento, data = DSL_M25_simca)

Linear Hypotheses:
          Estimate Std. Error t value Pr(>|t|)
HD - C == 0   2.7123     1.7942   1.512   0.3084
LD - C == 0   3.3667     1.4353   2.346   0.0757 .
LD - HD == 0   0.6545     1.6444   0.398   0.9162
---
```

```
> AnovaModel.7 <- aov(MUFA ~ trattamento, data=DSL_M25_simca)

> summary(AnovaModel.7)
            Df Sum Sq Mean Sq F value Pr(>F)
trattamento  2   82.1   41.06   0.617  0.551
Residuals   17 1131.9   66.58

> with(DSL_M25_simca, numSummary(MUFA, groups=trattamento,
+   statistics=c("mean", "sd")))
      mean        sd data:n
C 17.31323  4.851955      6
HD 19.88096 14.671742      4
LD 14.69904  6.397993     10

Fit: aov(formula = MUFA ~ trattamento, data = DSL_M25_simca)

Linear Hypotheses:
          Estimate Std. Error t value Pr(>|t|)
HD - C == 0    2.568      5.267   0.488    0.877
```

```
LD - C == 0   -2.614      4.214  -0.620    0.810
LD - HD == 0   -5.182      4.827  -1.073    0.541
```

> AnovaModel.12 <- aov(X.**UFA** ~ trattamento, data=DSL_M25_simca)

```
> summary(AnovaModel.12)
            Df Sum Sq Mean Sq F value Pr(>F)
trattamento  2  100.0   49.98   2.107  0.152
Residuals   17  403.2   23.72
```

> with(DSL_M25_simca, numSummary(X.**UFA**, groups=trattamento,
+   statistics=c("mean", "sd")))

```
      mean       sd data:n
C 51.33229 2.776968      6
HD 51.81999 7.162467      4
LD 55.98598 4.839500     10

Fit: aov(formula = X.UFA ~ trattamento, data = DSL_M25_simca)

Linear Hypotheses:
           Estimate Std. Error t value Pr(>|t|)
HD - C == 0   0.4877     3.1438   0.155    0.987
LD - C == 0   4.6537     2.5150   1.850    0.182
LD - HD == 0   4.1660     2.8813   1.446    0.339
```

> AnovaModel.10 <- aov(X.**SFA** ~ trattamento, data=DSL_M25_simca)

```
> summary(AnovaModel.10)
            Df Sum Sq Mean Sq F value Pr(>F)
trattamento  2  100.0   49.98   2.107  0.152
Residuals   17  403.2   23.72
```

> with(DSL_M25_simca, numSummary(X.**SFA**, groups=trattamento,
+   statistics=c("mean", "sd")))

```
      mean       sd data:n
C 48.66771 2.776968      6
HD 48.18001 7.162467      4
LD 44.01402 4.839500     10

Fit: aov(formula = X.SFA ~ trattamento, data = DSL_M25_simca)

Linear Hypotheses:
           Estimate Std. Error t value Pr(>|t|)
HD - C == 0  -0.4877     3.1438  -0.155    0.987
LD - C == 0  -4.6537     2.5150  -1.850    0.182
LD - HD == 0  -4.1660     2.8813  -1.446    0.338
```
